# Maternal Anemia and Pediatric Neurodevelopment in Children from Mothers Exposed to Mixed Heavy Metals in Suriname

**DOI:** 10.3390/jox16030110

**Published:** 2026-06-11

**Authors:** Anisma R. Gokoel, Arti Shankar, Simran F. Mokiem, Ashna D. Hindori-Mohangoo, Maureen Y. Lichtveld, Jeffrey K. Wickliffe, Wilco C. W. R. Zijlmans

**Affiliations:** 1 Faculty of Medical Sciences, Anton de Kom University of Suriname, Paramaribo, Suriname; wilco.zijlmans@uvs.edu; 2 Department of Environmental Health Sciences, School of Public Health and Tropical Medicine, Tulane University, New Orleans, LA 70112, USA; sarti@tulane.edu; 3 Foundation for Perinatal Interventions and Research in Suriname (Perisur), Paramaribo, Suriname; ashna.mohangoo@perisur.org; 4 School of Public Health, University of Pittsburgh, Pittsburgh, PA 15261, USA; mlichtve@pitt.edu; 5 Department of Environmental Health Sciences, School of Public Health, University of Alabama, Birmingham, AL 35294, USA; jkwickli@uab.edu

**Keywords:** maternal anemia, neurodevelopment, heavy metals, Suriname, BSID-III, environmental exposure, pregnancy

## Abstract

Maternal anemia and prenatal exposure to neurotoxic metals are widespread in low- and middle-income countries and may affect early childhood development. In Suriname, where mercury contamination from artisanal gold mining and social disparities coexist, we examined associations between maternal anemia, prenatal exposure to mercury (Hg), lead (Pb), manganese (Mn), selenium (Se), and cadmium (Cd), and early neurodevelopment. The study included 755 pregnant women and 644 children (10–26 months) from the Caribbean Consortium for Research in Environmental and Occupational Health (CCREOH) cohort. Maternal anemia was defined using WHO criteria for Hb, metals were measured in maternal blood, and child development was assessed using the Bayley Scales of Infant and Toddler Development, third edition (BSID-III-NL). Analyses used non-parametric tests, correlations, and multivariable regression. Anemia, though common (34%), was not independently associated with cognitive, language, motor, or social–emotional outcomes. However, iron status was not directly measured; therefore, the absence of an observed association should not be interpreted as evidence that maternal iron deficiency is unrelated to early neurodevelopment. Pb showed the most consistent associations, with higher prenatal levels predicting poorer cognitive, motor, and language scores. Hg demonstrated weaker but significant negative associations with several domains, while Mn and Cd showed limited direct effects. Interaction analyses suggested a potential modifying role of Se in certain metal-neurodevelopment associations; however, these findings require confirmation in future studies. Overall, these results suggest that prenatal exposure to neurotoxic metals and sociodemographic disparities may be important contributors to variation in early neurodevelopment in this population, but causal inferences cannot be made from this cross-sectional analysis.

## 1. Introduction

Anemia in pregnant women may have a detrimental effect on pediatric neurodevelopment. The fetal brain starts developing during early gestation, making its development particularly vulnerable to exposure, both chemical and non-chemical. In particular, low maternal iron status may lead to pediatric neurocognitive impairment [1]. Fetal iron deficiency has been demonstrated to compromise iron-dependent processes in the brain, including monoamine neurotransmission, neuronal growth and differentiation, myelination, and gene expression. These effects are both acute and long-term, extending into adulthood [2]. Mothers diagnosed with anemia during early pregnancy may have an increased risk of neurodevelopmental disorders in their offspring, such as autism spectrum disorder, attention-deficit/hyperactivity disorder, and intellectual disability (ID) [3,4]. Infants with iron deficiency have compromised recognition memory, slower processing, and poorer bonding that persist despite postnatal iron repletion [2]. This neurocognitive setback may persist throughout adolescence [5].

Childhood anemia is the world’s second leading cause of pediatric disability, and worldwide prevalence is highest among children under five years (39.8%) [6]. Among pregnant women global prevalence of anemia was 36.5% [7]. The highest prevalence (60%) was found among pregnant women living in low- and middle-income countries (LMICs), with the highest occurrence observed in tropical regions such as Sub-Saharan Africa, South Asia, the Caribbean, and Oceania [8]. A situation analysis on anemia in Latin America and the Caribbean from 1989 to 2009, conducted by the Pan American Health Organization (PAHO), revealed that the average subregional prevalence of anemia in preschool children aged 6–59 months is 33.9% in Mexico and Central America, 46.2% in South America, and 42.9% in the Caribbean. The average prevalence of anemia among nonpregnant women of childbearing age is 22.5% (16.3% in Mexico and Central America, 24.2% in South America, and 29.0% in the Caribbean). For pregnant women, the weighted average is 30.9% (21.3% in Mexico and Central America, 34.5% in South America, and 42.5% in the Caribbean) [9].

As such, anemia is a major public health problem in LMIC’s with significant adverse health consequences and a great impact on social and economic development [10].

For Suriname, a LMIC located on the north-eastern coast of South America, the World Health Organization reports an overall 26.9% mild-to-severe anemia prevalence rate among pregnant women in 2023. However, the Caribbean Consortium for Research in Environmental and Occupational Health (CCREOH; MekiTamara study [11]) reported a prevalence of 55% (unpublished results). A study in the remote tropical rainforest interior of Suriname reveals a high prevalence of anemia among the female population, with a reported 63% of women affected (Medical Mission Primary Health Care Suriname, unpublished results). There are several methods to measure anemia; however, the WHO report did not specify the hemoglobin (Hb) measurement method or sampling approach used to derive this estimate. Similarly, WHO reported overall mild-to-severe anemia prevalence rates for children 6–59 months (25.9%) [6], considerably lower than what was found in peers living in the interior of Suriname (63%) [12].

The Republic of Suriname has an ethnically diverse society, mainly consisting of Hindustanis, Creoles, Javanese, Tribal, Indigenous, and individuals of mixed ethnicity. The majority of its over half a million inhabitants live in the northern coastal plain. The far greater part of the country’s interior is covered by tropical rainforest and is inhabited primarily by Indigenous and Tribal peoples. In certain regions in the interior, there is artisanal and small-scale gold mining (ASGM) activity that makes use of elemental Hg during the gold extraction process. Much of this Hg is lost to the environment and, once converted to methylmercury, enters the food chain and piles up in predatory fish. In the interior, people rely heavily on fish consumption for their daily protein requirement and consume these often more than once daily, including pregnant women [13,14]. Both total and methylmercury levels found in pregnant women living in the interior were significantly higher compared to pregnant women living in the coastal area [15]. Prenatal exposure to certain environmental pollutants, such as Hg, Pb, and Mn, is positively associated with increased adverse pediatric health outcomes and developmental delay [16,17]. Se plays a crucial role in pregnancy, supporting both maternal and fetal cognitive development. Because environmental exposures rarely occur in isolation, Se was examined not only as an independent exposure but also for its potential interaction with toxic metals, particularly Hg, based on evidence that Se may influence metal bioavailability and toxicity [18,19,20]. Previous studies have reported that Hg exposure was particularly harmful at low Se levels, while higher Se levels seemed to mitigate the effects of Hg [18,19,20].

This study assesses the influence of anemia in CCREOH mothers exposed to different concentrations of Hg, Pb, Mn, and Se on pediatric neurodevelopment. Neurodevelopment is measured using the Bayley Scales of Infant and Toddler Development (BSID-III-NL), which has been found to be valid and reliable in assessing the neurodevelopment of infants in Suriname [21]. We hypothesize that children of CCREOH mothers with moderate or severe anemia have lower BSID scores than children from non-anemic mothers.

Second, we hypothesize that prenatal heavy metal exposure is associated with reduced BSID scores.

## 2. Materials and Methods

### 2.1. Study Population

Mother/child dyads were selected from the Caribbean Consortium in Environmental and Occupational Health (CCREOH) cohort, an environmental epidemiological study conducted in the Republic of Suriname, South America [11]. During the first or second trimester of pregnancy, pregnant women were recruited in three regions of Suriname: Paramaribo, the Amazonian Interior, and Nickerie, where different types and levels of exposures of both non-essential and essential elements are expected due to differences in diets and metal-based pesticides [11]. All included women (18+) provided written informed consent, and assent was obtained from participants who were 16 or 17 years old. The inclusion of 1200 pregnant women and their singleton birth children was requested and approved by both the Institutional Review Board of the Government of Suriname and Tulane University [11]. Exclusion criteria for the infants include the following: (1) birth before 33 completed weeks of gestation and/or weighing less than 2000 g at birth; (2) the presence of certain congenital anomalies, such as Down syndrome, hydrocephalus, and medical conditions such as cerebral palsy [11]. The study included 755 pregnant women, of whom 644 children aged 10–26 months were included in the present analysis. A total of 111 children were not included due to loss to follow-up resulting from relocation or withdrawal from the study.

### 2.2. Maternal Anemia

The World Health Organization (WHO, 2025) defines anemia as a condition in which the number of red blood cells or their oxygen-carrying capacity is insufficient to meet the body’s physiologic needs, which vary by age, sex, altitude, smoking, and pregnancy status [22]. The WHO’s published Hb cut-off point defines severe (<4.3 mmol/L), moderate (4.3–6.2 mmol/L), and mild (6.2–6.8 mmol/L) anemia [23]. In the current study, maternal anemia was considered the main predictor of infant/toddler’s neurodevelopment and studied as both continuous and categorical variables based on the WHO cut-offs. Moderate and severe anemia were combined into a single category due to the small number of severe anemia cases (1.7%).

### 2.3. Neurodevelopment Scaled Scores—BSID-III

The Bayley Scales of Infant and Toddler Development, 3rd edition (BSID-III) was used to determine neurodevelopment and was completed between 11 and 42 months of age. The BSID-III is a widely utilized, individually administered, age-standardized instrument designed to assess the developmental functioning of infants and toddlers from 1 month to 42 months of age. It is employed to identify possible neurodevelopmental delay or disabilities [24]. The BSID-III was created for United States children, validated for use in the Netherlands (BSID-III-NL), and later found to be valid and reliable for use on Surinamese infants. A total of 299 children from the three geographic locations mentioned above, between the ages of 10–26 months, were included in the study by McLester-Davis, L.W.Y. et al. [21].

The BSID-III is a comprehensive assessment tool designed to evaluate the developmental progress of infants and toddlers across multiple domains, including cognitive, language, motor, and social–emotional abilities. The assessment of the social–emotional domain is conducted through the administration of a questionnaire to the primary caregiver. Testing was performed by a team of psychologists and trained assistants, with oversight from a psychiatrist. The age-specific start point prior to administration was determined by calculating the chronological age. The administration and interpretation of the Bayley test were conducted in accordance with the detailed manual instructions [25]. After the assessment, the examiner summarized the raw score of each domain, converted it to scaled scores, and verified the scores before entering in the database. Cognitive, gross and fine motor, receptive and expressive communication, and social–emotional were the six neurodevelopment scaled scores that were assessed as continuous outcome variables.

### 2.4. Environmental Contaminants and Analysis

Maternal biospecimens were collected at two time points during pregnancy: late first/early second, and third trimester; postpartum assessments were conducted for both mother and child until 48 months. For the present analysis, the first measured maternal metal exposure value obtained during the late first or early second trimester was used, as it corresponded with the timing of the Hb assessment. Maternal blood samples were tested for trace elements Hg, Pb, Cd, and Mn. These samples were sent to the Wisconsin State Laboratory of Hygiene Trace Element Research Laboratory and the Wadsworth Center, and were tested using inductively coupled plasma-mass spectrometry or cold-vapor atomic fluorescence spectrometry (CVAFS) [11]. The trace metals were evaluated as continuous variables. Although both maternal Hb concentrations and metal exposure levels may fluctuate throughout pregnancy, the use of the first available measurement allowed temporal alignment with the Hb assessment.

### 2.5. Maternal Sociodemographics

Covariates included age at study intake, parity, marital status, household income, educational level, and ethnic background. Age was examined continuously. Parity was categorized as zero to one vs. two or more, depending on the number of prior live births.

Ethnic background, based on self-report, was categorized based on the main ethnic groups in Suriname (Creole, Hindustani, Indigenous, Javanese, Tribal People, and Mixed). Educational level was based on the participants’ highest completed degree and categorized as (1) not educated and primary education, (2) lower secondary education/vocational training, and (3) upper secondary education/vocational training and tertiary education. An analysis of household income was conducted, with data categorized into four subgroups based on monthly household income in Surinamese Dollars (SRD): <1500 vs. 1500–2999 vs. 3000–4999 vs. 5000+ SRD. Marital status was dichotomized into the categories married or living with a partner vs. unmarried or not living with a partner.

### 2.6. Statistical Analyses

Descriptive statistics of the study population were calculated and presented stratified by subgroups of maternal anemia. Associations between maternal sociodemographics and subgroups of maternal anemia were tested using the Chi-squared-test for categorical variables. Non-parametric analyses were conducted using the Mann–Whitney U test for comparisons between two groups and the Kruskal–Wallis test for comparisons involving more than two groups. These analyses were used to examine the association between the neurotoxicants and anemia or each of the neurodevelopmental scaled scores. Due to non-linear relations between blood metals and neurodevelopmental scaled scores, Spearman’s rho was used to assess for associations. To assess potential effect modification between blood metals and anemia on neurodevelopmental outcomes, stratified analyses and interaction models were performed. Multivariable linear regression models were applied to determine whether exposure–outcome associations varied across strata of other elements. Regression assumptions and multicollinearity were assessed prior to conducting the analyses. Although the assumption of normality was not fully met, linear regression was considered appropriate given the relatively large sample size (n = 644) and the robustness of regression methods to moderate departures from normality. Potential multicollinearity among metal exposure variables was assessed using Spearman correlation coefficients. Correlations between metals were moderate (e.g., Pb–Hg: ρ = 0.55, Pb–Se: ρ = 0.41, Hg–Se: ρ = 0.46) and did not indicate severe multicollinearity. Regression coefficients and standard errors were stable across models, suggesting that multicollinearity was not a major concern.

All analyses were performed using the IBM Statistical Package for the Social Sciences (SPSS) software version 28 for Windows.

## 3. Results

Maternal Hb levels ranged from 3.2 to 12.9 mmol/L and were normally distributed (mean ± standard deviation 6.6 ± 1.0 mmol/L). The prevalence of maternal anemia based on the WHO definition of Hb < 6.2 mmol/L was 34%. General characteristics of the study population, stratified for subgroups of maternal anemia, are presented in Table 1. Maternal age (*p* = 0.483) and marital status (*p* = 0.368) were not significantly associated with maternal anemia, whilst parity (*p* = 0.037), ethnic background (*p* < 0.001), educational level (*p* = 0.007), and household income (*p* = 0.024) showed a significant association. Higher proportions of anemia were observed among participants with two or more previous live births (38% vs. 31% for those with lower parity) and among Creole (49%) and Tribal (41%) participants. The majority of the participants with a lower secondary or vocational education (69%) had moderate or severe anemia. The highest prevalence of moderate or severe anemia was observed in the 1500–2999 SRD household income group (40%). The distribution of blood Hg values differed significantly among subgroups of maternal anemia. Participants with moderate or severe maternal anemia had significantly lower median Hg values compared with participants with mild or no anemia (2.55 vs. 3.30 μg/L; *p* < 0.001). Participants with moderate or severe anemia had significantly higher median Cd (0.22 vs. 0.18 μg/L; *p* < 0.001) and median Mn (14.40 vs. 12.80 μg/L; *p* = 0.004) values compared with participants with mild or no anemia.

The distribution of neurodevelopment scaled scores by maternal sociodemographic factors is presented in Table 2. Parity was significantly associated with cognitive (*p* = 0.017), expressive (*p* = 0.037), and social–emotional (*p* = 0.037) scaled scores. Children of mothers with 0–1 previous live births had higher mean ranks in these domains compared to those with two or more previous live births, suggesting that higher parity is associated with lower Bayley scores. Ethnic background was significantly associated with cognitive (*p* = 0.018), fine motor (*p* = 0.023), gross motor (*p* < 0.001), expressive communication (*p* < 0.001), and social–emotional (*p* = 0.011) scores, suggesting variation in developmental outcomes across ethnic groups. Across most Bayley scales, children from Tribal and Indigenous populations showed the lowest scores. Maternal educational level showed significant associations with cognitive (*p* = 0.008), fine motor (*p* = 0.013), gross motor (*p* < 0.001), receptive communication (*p* = 0.002), and expressive communication (*p* < 0.001) scores, with higher educational levels associated with higher developmental scores. Household income was significantly associated with better cognitive (*p* = 0.043), gross motor (*p* < 0.001), and expressive communication (*p* < 0.001) scores. Higher mean ranks were noted with a higher household income, suggesting that a higher household income is associated with better neurodevelopment scores. Maternal anemia and marital status showed no significant associations with any of the developmental domains.

Table 3 shows the association between blood metal levels and neurodevelopmental outcomes. Higher blood Pb levels were significantly associated with reduced scores on the cognitive (ρ = −0.099, *p* = 0.012), fine motor (ρ = −0.085, *p* = 0.031), gross motor (ρ = −0.192, *p* < 0.001), receptive communication (ρ = −0.112, *p* = 0.005), and expressive communication (ρ = −0.206, *p* < 0.001) domains. Blood Hg levels showed weak negative associations with gross motor (ρ = −0.124, *p* = 0.002), expressive communication (ρ = −0.117, *p* = 0.003), and social–emotional (ρ = −0.093, *p* = 0.018) scores. Se concentrations also showed weak negative associations with most domains: cognitive (ρ = −0.088, *p* = 0.026), gross motor (ρ = −0.117, *p* = 0.003), receptive communication (ρ = −0.132, *p* = 0.001), and expressive communication (ρ = −0.164, *p* < 0.001). A weak positive, but statistically significant association was found between blood Cd levels and gross motor (ρ = 0.095, *p* = 0.016) and expressive communication (ρ = 0.126, *p* = 0.001). No significant associations were found between blood Mn levels and any of the Bayley domains.

**Figure 1:**
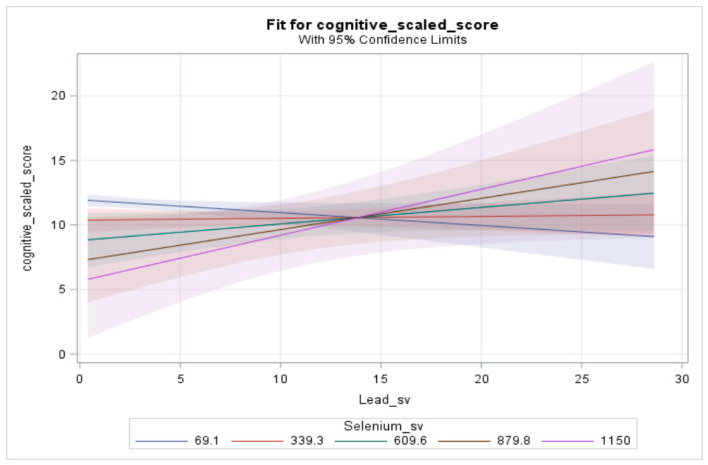
Association of Pb level and cognitive score: the role of Se.

**Figure 2:**
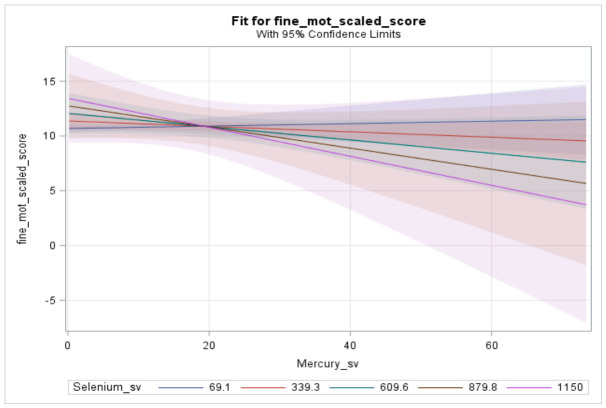
Association of Hg and fine motor score: the role of Se.

**Figure 3:**
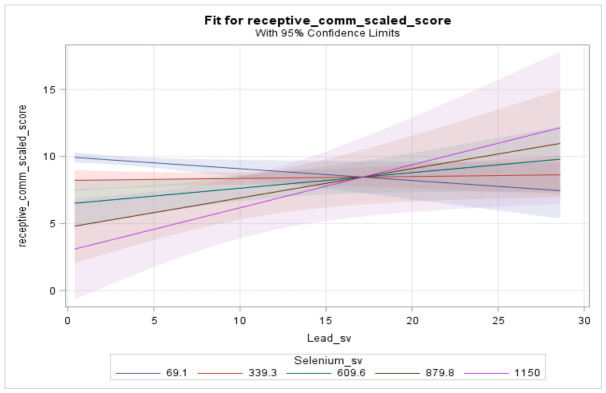
Association of Pb and receptive communication score: the role of Se.

Figure 1, Figure 2 and Figure 3 show the cumulative exposure of environmental contaminants on neurodevelopment. Figure 1 shows that in mothers with low Se levels, cognitive scores decreased with increasing Pb levels. In mothers with high Se levels, cognitive scores increased with increasing Pb levels.

In mothers with lower Se levels, fine motor scores increased with increasing Hg levels, while in mothers with higher Se levels, fine motor scores decreased with increasing Hg levels (see Figure 2).

Figure 3 shows that in mothers with low Se levels, the receptive communication scores decreased with increasing Pb levels. In mothers with high Se levels, the receptive communication scores increased with increasing Pb levels.

**Figure 4:**
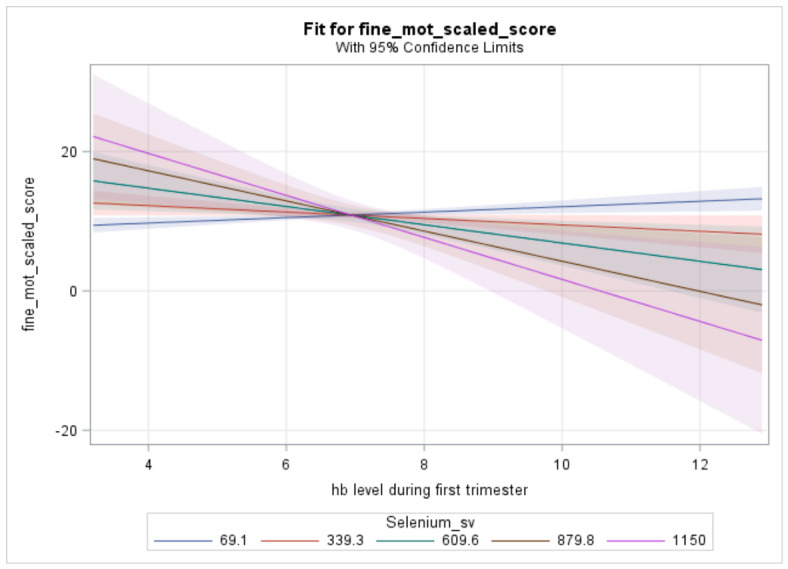
Association of Hb and fine motor score: the role of Se.

**Figure 5:**
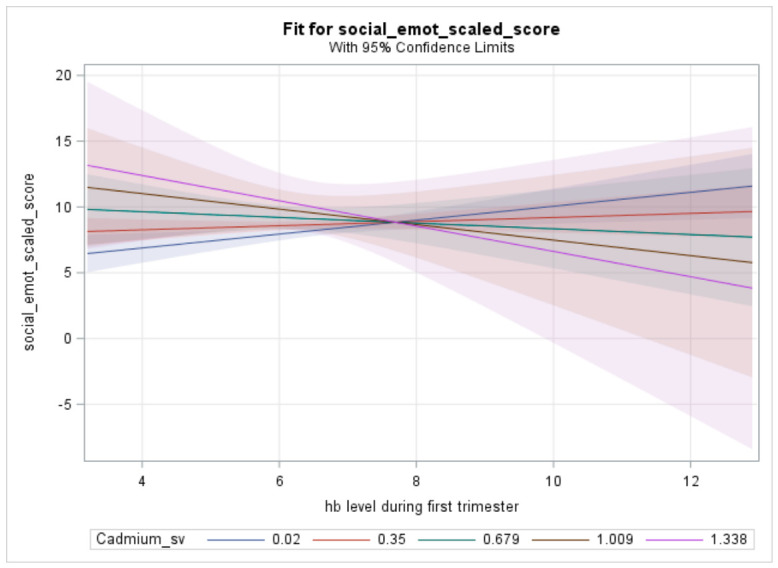
Association of Hb and social emotional score: the role of Cd.

Figure 4 and Figure 5 show the association between anemia and elements on neurodevelopment using BSIDIII. In mothers with low Se levels, fine motor scores increased with increasing Hb levels. In mothers with high Se levels, fine motor scores decreased with increasing Hb levels (see Figure 4).

In mothers with low Cd levels, social–emotional scores increased with increasing Hb levels. In mothers with high Cd levels, social–emotional scores decreased with increasing Hb levels (see Figure 5).

In the regression analysis (Table 4), none of the main exposures—anemia, Hg, Pb, Cd, Mn, or Se—were individually associated with cognitive scores. However, a significant positive interaction was found between Pb and Se (Pb × Se: β = 0.00062, SE = 0.00027, *p* = 0.0215), indicating that the association between prenatal Pb exposure and cognitive development varied by Se levels (β = −2.84, SE = 0.91, *p* = 0.0018).

Specifically, higher Se concentrations attenuated the negative association between Pb exposure and cognitive scores. In contrast, no significant interactions were observed between Se and Hg, Cd, or Mn. Demographic and maternal covariates were not significantly associated with cognitive outcomes in the adjusted model.

In the regression analysis (Table 5), none of the main exposures (Hg, Pb, Cd, Mn, or Se) were independently associated with fine motor scores. However, a significant negative interaction was observed between Hg and Se (Hg × Se: β = −0.00031, SE = 0.00015, *p* = 0.0327), indicating that the association between prenatal Hg exposure and fine motor development varied according to Se levels. Specifically, higher Se concentrations appeared to modify the association between Hg exposure and fine motor scores in a negative direction. In contrast, no significant interactions were observed between Se and Pb, Cd, or Mn. Among covariates, lower household income (SRD < 800) and belonging to the Tribal ethnic group were associated with lower fine motor scores, while other demographic and maternal factors were not significantly associated.

In the regression analysis (Table 6), none of the main exposures (Hg, Cd, Mn, Se, or anemia categories) were independently associated with receptive communication scores. However, a significant positive interaction was observed between Pb and Se (Pb × Se: β = 0.00046, SE = 0.00022, *p* = 0.0373), indicating that the association between prenatal Se exposure and receptive communication varied by Se levels. Higher Se concentrations appeared to modify the association between Pb exposure and receptive communication outcomes. In contrast, no significant interactions were observed between Se and Hg, Cd, or Mn. Among covariates, higher household income (SRD ≥ 800) was associated with lower receptive communication scores, while other demographic and maternal factors were not significantly associated.

In analyses examining gross motor, expressive communication, and social–emotional outcomes, none of the main exposures—anemia, Hg, Pb, or Se—were significant predictors. Additionally, there were no significant interaction effects between Pb and Se or between Hg and Se for any of these outcomes.

## 4. Discussion

This study examined the association between maternal anemia and early childhood neurodevelopmental outcomes in a multi-ethnic cohort of Surinamese mother–child dyads exposed to varying levels of contaminants. Previous studies have shown that low maternal Hb levels and anemia during pregnancy were associated with reduced psychomotor and neurocognitive scores [27,28]. However, despite the high prevalence of moderate and severe maternal anemia in our cohort (34.0% of pregnant women), maternal anemia was not associated with lower BSID-III neurodevelopmental scores in children. The etiology of anemia was not explored in this study. This may reflect a limitation of using Hb concentration alone as an indicator of iron status, as Hb cannot distinguish iron-deficiency anemia from anemia due to other causes, including inflammation, hemoglobinopathies, or micronutrient deficiencies. Iron deficiency during pregnancy has a well-established link with neurodevelopmental delay [2,5]. Independent of iron deficiency, conditions such as hemoglobinopathies may themselves affect neurodevelopment [29]. The use of Hb as a proxy for iron status, together with the presence of other causes of anemia, may have attenuated the observed association between maternal anemia and child neurodevelopment. These findings highlight the need for a more comprehensive assessment of maternal biomarkers during pregnancy to better elucidate this relationship. We observed significant associations between maternal factors—including parity, ethnicity, and education—and neurodevelopmental domains. Higher parity was associated with lower neurodevelopmental scores, consistent with a study in Bangladesh in 9453 children aged from 36 to 59 months, suggesting resource dilution in larger families [30]. Ethnic background showed strong associations with all neurodevelopmental domains except for receptive communication. Children of Indigenous and Tribal mothers had lower scores overall, particularly in gross motor and expressive communication domains. This finding may reflect socioeconomic disparities, environmental stressors, cultural testing bias, or differences in child-rearing practices. Higher maternal education correlated with better performance across nearly all domains, consistent with a study by Ye et al. in China [31].

Prenatal exposure to heavy metals, particularly Pb, Hg, and Se, was negatively associated with several domains of neurodevelopment. Pb was most strongly associated with expressive communication deficits (ρ = −0.206), consistent with the existing literature on Pb’s neurotoxic impact on language and cognition [32,33]. A notable finding in this study is the absence of an association between Pb exposure and anemia. Although Pb is a well-established cause of anemia [34], such effects are typically observed at higher exposure levels (>5 μg/dL) [35] compared to those identified in our population. In addition, the Mann–Whitney U test revealed no statistically significant difference in Pb levels between the moderate/severe anemia group and the mild/no anemia group (U = 63,419.5, Z = −0.03, *p* = 0.976), with nearly identical mean ranks across groups. These findings suggest that the levels of Pb exposure in this Surinamese cohort may be insufficient to elicit hematological effects, including anemia.

Hg showed weaker but significant associations with motor and social–emotional development, aligning with the known neurotoxic effects of methylmercury exposure from fish consumption in Suriname’s interior. Similar findings have been reported in previous studies, including associations between prenatal Hg exposure and reduced motor skills at 18 months in Croatia [36], as well as impaired gross motor function and reduced social interaction in children in Tanzania [37].

The observed interaction patterns suggest that Se may modify the relationship between prenatal metal exposures and early neurodevelopment; however, these effects were heterogeneous across metals and developmental domains and should be interpreted cautiously. From a biological perspective, established evidence indicated that Hg and Se are closely interconnected, with Se capable of binding Hg and reducing its toxicity through the formation of inert complexes [38]. Both elements often originate from similar dietary sources, particularly fish consumption, which may result in correlated exposure [39,40]. Previous studies have reported weak positive correlations between blood Hg and Se concentrations, suggesting that higher Se levels may co-occur with higher Hg exposure [41,42]. Se is shown to exert a protective role against Hg toxicity, although this effect may depend on exposure levels and the Se-to-Hg ratio [43].

Within this established context, the statistical interaction analyses in our study may be explained by two plausible theories for the observed adverse effects of Se. First, the negative associations observed in our study may not indicate a direct adverse effect of Se, but could be attributable to residual confounding due to co-exposure with Hg. Although Se has a known protective role against Hg toxicity, this effect may be insufficient at higher exposure levels or may depend on the Se–Hg ratio, as seen in the previous literature [43]. Second, a U-shaped relationship between Se and neurodevelopmental outcomes has been proposed in the previous literature, where both low and high levels are associated with suboptimal cognitive performance [19]. Se concentrations ≥ 250 ug/mL have been linked to adverse effects [39], and in our study, 10% of mothers exceeded this threshold.

Our findings that higher Se levels strengthened the adverse association between Pb exposure and cognitive development as well as receptive communication are consistent with evidence from prenatal studies showing complex metal–metal interactions. For example, Shih et al. (2021) reported synergistic interactions between Se and Pb, as well as Se and Hg, in relation to birth outcomes, suggesting that Se does not universally confer protection and may, under certain exposure conditions, exacerbate toxicity [44]. These findings should be interpreted with caution, as the direction and presence of interactions were not consistent across metals or neurodevelopmental domains. While Se has been hypothesized to play a protective role against neurotoxic effects through antioxidant and binding mechanisms, the present results do not support a uniform protective effect across exposures. Instead, they suggest a more complex and context-dependent role, potentially reflecting differences in exposure mixtures, developmental timing, or susceptibility of specific neurodevelopmental domains. These results require confirmation in future studies with targeted designs and replication in independent cohorts.

In our cohort, maternal Cd concentrations were relatively low, with a median (IQR) of 0.20 µg/L (0.13–0.29 µg/L) and showed limited variability across participants. Placental transfer of Cd is restricted due to sequestration within the placenta, resulting in substantially lower fetal exposure relative to maternal concentrations [45,46]. A possible hypothesis is that maternal blood Cd may not directly reflect the biologically relevant dose to the developing fetal brain. Instead, the observed associations may reflect maternal Cd levels as a proxy marker of correlated factors such as dietary patterns or socioeconomic conditions, or other environmental exposures that are more strongly associated with child neurodevelopment [47]. Accordingly, the weak positive associations observed in this study should not be interpreted as causal but may instead reflect residual confounding or proxy effects rather than a beneficial effect of Cd. These findings emphasize the need for integrated approaches that consider co-exposures, nutritional status, and maternal sociodemographic factors when assessing early childhood risk.

No associations were observed between Mn concentrations and neurodevelopmental outcomes. Mn levels are known to vary across pregnancy, typically increasing in later gestation [48,49,50], with the second trimester representing a critical exposure window [45]. This pattern was also observed in our cohort. Although measurements were available across all three trimesters, the majority (89%) were obtained during the first and second trimesters, corresponding to the timing of Hb assessment. While this approach allowed alignment between Hb and metal exposure assessments, it may have limited our ability to capture variability in Mn exposure later in pregnancy.

This study has several notable strengths. First, it leverages a large and ethnically diverse sample from a LMIC setting, which enhances the generalizability of the findings to similar populations. Second, the neurodevelopmental outcomes were assessed using the BSID-III, a well-validated and culturally adapted tool for Surinamese infants, which strengthens the reliability of the developmental assessments. Third, the study comprehensively examined multiple maternal, environmental, and social determinants, including heavy metal exposure, allowing for a nuanced understanding of the multifactorial influences on child neurodevelopment. This is one of the few studies performed on blood metals and neurodevelopment in the Caribbean.

Our study also has limitations. The use of cross-sectional neurodevelopmental assessments limits the ability to establish causality or examine developmental trajectories over time. The interaction terms included in our models were specified a priori based on biological plausibility; interaction analyses generally require greater statistical power and should therefore be interpreted with appropriate caution. Also, the metal measurements in the mothers were one-time assessments, and the study did not consider whether anemic mothers received iron supplements, which could have affected the observed outcomes. In addition, information about the children’s exposure to metals after birth is lacking, as their diet may have influenced our findings. Maternal nutritional factors and postnatal environmental exposures, which are important determinants of anemia and child neurodevelopment, were collected as part of the CCREOH study but were beyond the scope of the present analysis. Residual confounding related to maternal nutritional status, postnatal environmental exposures, socioeconomic factors, and other unmeasured developmental influences cannot be excluded.

## 5. Implications for Research, Policy, and Practice

These results highlight the multifactorial nature of neurodevelopmental outcomes in LMIC contexts. While maternal anemia alone may not be a dominant predictor, its interaction with environmental toxicants and sociodemographic disparities plays a critical role. Public health efforts should prioritize the following:

- Early screening and treatment of anemia during pregnancy.
- Reducing exposure to neurotoxic metals; for Suriname, this would particularly regard mercury in the interior.
- Enhancing maternal education and supporting high-risk ethnic groups. This includes both general education—access to quality schooling to improve health literacy—and specific health education—culturally tailored counseling on nutrition, anemia prevention, and reducing toxicant exposure.
- Continuing education for primary care physicians, midwives, and nurse aides practicing on the prenatal care frontline, especially those providing prenatal care in the interior region.
- Designing follow-up studies to track long-term cognitive and behavioral outcomes. Importantly, given that neurodevelopment was assessed at an early age (10–42 months), observed effects may still be subtle and not fully captured at this developmental stage. Differences may become more apparent with increasing age and with the use of more sensitive, age-appropriate assessments. Ongoing longitudinal follow-up of the CCREOH cohort, including school-age neurodevelopmental assessments and expanded biomarker profiling, is therefore essential to better understand the long-term impact of prenatal exposures and to better inform targeted interventions.

## Figures and Tables

**Table 1:** General characteristics of the study population stratified by subgroups of maternal anemia.

	Maternal Anemia					
	Moderate or Severe		Mild or No			
	<6.2 mmol/L		6.2+ mmol/L		Total	*p*-Value
	n (253)	%	n (502)	%	n	
	253	34%	502	66%	755	
Age						0.483
Mean ± SD	27.8 ± 6.2		28.1 ± 6.3		755	
Parity						**0.037**
0–1 previous live births	137	31%	312	69%	449	
2+ previous live births	115	38%	189	62%	304	
Total	252	33%	501	67%	753	
Ethnic background						**<0.001**
Creole	83	49%	85	51%	168	
Hindustani	44	29%	107	71%	151	
Indigenous	12	13%	82	87%	94	
Javanese	10	18%	46	82%	56	
Tribal people	58	41%	82	59%	140	
Mixed	46	32%	100	68%	146	
Total	253	34%	502	66%	755	
Educational level						**0.007**
primary or not	49	28%	129	72%	178	
lower secondary/vocational	102	41%	147	59%	249	
upper secondary/vocational and tertiary	102	31%	226	69%	328	
Total	253	34%	502	66%	755	
Household income in SRD *						**0.024**
<1500	82	30%	189	70%	271	
1500–2999	92	40%	137	60%	229	
3000+	69	30%	164	70%	233	
Total	243	33%	490	67%	733	
Marital status						0.368
Married/living together	218	33%	444	67%	662	
Unmarried/single	35	38%	58	62%	93	
Total	253	34%	502	66%	755	
Blood metal level						
Hg median [IQR] μg/L	2.55 [1.52–4.71]		3.30 [1.95–6.62]		755	**<0.001**
Pb median [IQR] μg/dL	1.98 [1.30–3.10]		1.86 [1.21–3.85]		755	0.976
Se median [IQR] μg/L	146.00 [111.50–196.97]		145.50 [110.82–203.19]		755	0.641
Cd median [IQR] μg/L	0.22 [0.15–0.32]		0.18 [0.12–0.26]		755	**<0.001**
Mn median [IQR] μg/L	14.40 [10.81–18.79]		12.80 [10.20–16.51]		755	**0.004**

* SRD = Surinamese dollar, equivalent to 0.38 USD [26]. IQR = Interquartile Range. Bold indicates significance.

**Table 2:** Distribution of Bayley scaled scores for maternal anemia and maternal sociodemographics.

	Bayley Scale Scores (Mean Rank)					
	N = 644					
	Cognitive	Fine Motor	Gross Motor	Receptive Communication	Expressive Communication	Social–Emotional
Maternal anemia						
Moderate and severe (n = 208)	310.45	310.89	325.67	317.93	316.87	303.78
Mild or no (n = 436)	328.25	328.04	320.99	324.68	325.19	331.43
*p*-value	0.254	0.270	0.764	0.664	0.592	0.075
Parity						
0–1 previous live births (n = 390)	335.95	325.47	330.81	322.35	334.20	334.20
2+ previous live births (n = 253)	300.50	316.64	308.41	321.46	303.19	303.19
*p*-value	**0.017**	0.553	0.132	0.952	**0.037**	**0.037**
Ethnic background						
Creole (n = 138)	312.70	337.99	339.22	320.39	338.88	302.42
Hindustani (n = 138)	350.71	324.96	361.67	351.25	360.58	358.98
Indigenous (n = 78)	307.96	319.69	217.96	288.97	211.18	321.83
Javanese (n = 47)	370.02	355.03	324.54	328.52	358.39	344.79
Tribal people (n = 118)	280.39	270.66	298.81	292.66	288.66	279.68
Mixed (n = 125)	333.14	341.15	347.62	339.93	350.29	336.86
*p*-value	**0.018**	**0.023**	**0.000**	0.070	**0.000**	**0.011**
Educational level						
primary or not (n = 145)	305.27	289.80	256.59	287.44	248.16	302.37
lower secondary/vocational (n = 208)	299.84	315.53	325.39	310.25	319.21	316.56
upper secondary/vocational and tertiary (n = 291)	347.28	343.78	353.27	348.73	361.89	336.77
*p*-value	**0.008**	**0.013**	**0.000**	**0.002**	**0.000**	0.157
Household income in SRD						
<1500 (n = 233)	296.53	291.97	267.44	295.76	267.11	314.47
1500–2999 (n = 191)	305.40	319.46	331.26	326.55	325.44	298.45
3000–4999 (n = 136)	341.06	334.36	350.74	326.99	353.57	323.57
5000+ (n = 68)	348.51	338.07	356.20	319.87	368.01	341.57
*p*-value	**0.043**	0.085	**0.000**	0.251	**0.000**	0.331
Marital status						
Married/living together (n = 567)	325.87	323.33	318.66	321.52	320.00	323.04
Unmarried/single (n = 77)	297.72	316.41	350.78	329.73	340.94	318.56
*p*-value	0.210	0.758	0.152	0.714	0.350	0.841

Bold indicates significance.

**Table 3:** Association between blood metal level and Bayley scaled scores.

Blood Metal Level	Bayley Scale Scores (Spearman’s Rho)					
	Cognitive	Fine Motor	Gross Motor	Receptive Communication	Expressive Communication	Social–Emotional
Hg	−0.036	0.008	−0.124	−0.059	−0.117	−0.093
*p*-value	0.360	0.837	**0.002**	0.134	**0.003**	**0.018**
Pb	−0.099	−0.085	−0.192	−0.112	−0.206	−0.032
*p*-value	**0.012**	**0.031**	**0.000**	**0.005**	**0.000**	0.419
Se	−0.088	−0.014	−0.117	−0.132	−0.164	−0.005
*p*-value	**0.026**	0.727	**0.003**	**0.001**	**<0.001**	0.890
Cd	0.005	−0.022	0.095	0.025	0.126	0.035
*p*-value	0.898	0.569	**0.016**	0.534	**0.001**	0.369
Mn	−0.036	−0.053	−0.001	−0.036	−0.026	0.031
*p*-value	0.357	0.183	0.985	0.366	0.505	0.432

Bold indicates significance.

**Table 4:** Multiple linear regression coefficients for predictors of cognitive development.

Parameter	Estimate	Standard Error	T-Value	*p*-Value	95% CI	
Intercept	11.84981	1.41412	8.38	<0.0001	9.072361	14.62726
Hg	0.043317	0.051592	0.84	0.4015	−0.05801	0.144647
Pb	−0.14391	0.086264	−1.67	0.0958	−0.31334	0.025524
Cd	−2.10878	3.21729	−0.66	0.5124	−8.42781	4.210246
Mn	0.003374	0.056416	0.06	0.9523	−0.10743	0.11418
Se	−0.005	0.005675	−0.88	0.3783	−0.01615	0.006142
Maternal age	−0.00311	0.023675	−0.13	0.8954	−0.04961	0.043387
Married	0.471749	0.416203	1.13	0.2575	−0.34571	1.289207
Not married or living with a partner *	0					
Education	0.208154	0.255474	0.81	0.4155	−0.29362	0.709926
Creole	−0.22455	0.39694	−0.57	0.5718	−1.00417	0.555073
Hindustani	0.211721	0.391638	0.54	0.589	−0.55749	0.980931
Indigenous	0.22783	0.64652	0.35	0.7247	−1.04199	1.49765
Javanese	0.825684	0.544478	1.52	0.1299	−0.24372	1.895084
Tribal	−0.52479	0.431353	−1.22	0.2242	−1.37201	0.32242
Mixed *	0					
Household income SRD 800+	−0.59628	0.391917	−1.52	0.1287	−1.36604	0.173478
Household income <SRD800 *	0					
0–1 previous live births	0.293807	0.311543	0.94	0.346	−0.31809	0.905704
2+ previous live births *	0					
Moderate anemia	−0.14168	0.298452	−0.47	0.6352	−0.72786	0.444508
Mild anemia	−0.00044	0.33989	0	0.999	−0.66801	0.667135
No anemia *	0					
Pb × Se	0.000618	0.000268	2.31	**0.0215**	9.14 × 10^−5^	0.001144
Hg × Se	−0.00023	0.000171	−1.32	0.1882	−0.00056	0.000111

* = reference category. Bold indicates significance.

**Table 5:** Multiple linear regression coefficients for predictors of fine motor development.

Parameter	Estimate	Standard Error	t-Value	*p*-Value	95% CI	
Intercept	10.1183	1.194392	8.47	<0.0001	7.772418	12.46419
Hg	0.068092	0.043575	1.56	0.1187	−0.01749	0.153678
Pb	−0.04611	0.072861	−0.63	0.527	−0.18922	0.096989
Cd	0.675935	2.717381	0.25	0.8036	−4.66123	6.013099
Mn	−0.01042	0.04765	−0.22	0.827	−0.10401	0.083169
Se	0.004136	0.004793	0.86	0.3886	−0.00528	0.01355
Maternal age	0.022124	0.019997	1.11	0.269	−0.01715	0.061399
Married	0.069258	0.351533	0.2	0.8439	−0.62118	0.759698
Not married or living with a partner *	0					
Education	0.314037	0.215778	1.46	0.1461	−0.10977	0.737843
Creole	−0.16258	0.335263	−0.48	0.6279	−0.82107	0.495902
Hindustani	−0.26243	0.330784	−0.79	0.4279	−0.91212	0.38726
Indigenous	−0.58618	0.546062	−1.07	0.2835	−1.65869	0.486337
Javanese	0.341742	0.459876	0.74	0.4577	−0.56149	1.244976
Tribal	−0.89922	0.364328	−2.47	**0.0139**	−1.61479	−0.18365
Mixed *	0					
Household income SRD 800+	−0.77164	0.33102	−2.33	**0.0201**	−1.42179	−0.12149
Household income <SRD800 *	0					
0–1 previous live births	0.179	0.263135	0.68	0.4966	−0.33782	0.695819
2+ previous live births *	0					
Moderate anemia	0.01939	0.252078	0.08	0.9387	−0.47571	0.514492
Mild anemia	0.064488	0.287077	0.22	0.8223	−0.49936	0.628331
No anemia *	0					
Pb × Se	0.000308	0.000226	1.36	0.1739	−0.00014	0.000753
Hg × Se	−0.00031	0.000145	−2.14	**0.0327**	−0.00059	−2.6 × 10^−5^

* = reference category. Bold indicates significance.

**Table 6:** Multiple linear regression coefficients for predictors of receptive communication scores.

Parameter	Estimate	Standard Error	t-Value	*p*-Value	95% CI	
Intercept	11.0112	1.15928	9.5	<0.0001	8.73432	13.2882
Hg	0.03294	0.04229	0.78	0.4364	−0.0501	0.11601
Pb	−0.1393	0.07072	−1.97	**0.0494**	−0.2782	−0.0004
Cd	−0.9105	2.63749	−0.35	0.73	−6.0908	4.26971
Mn	−0.0292	0.04625	−0.63	0.5281	−0.12	0.06164
Se	−0.0076	0.00465	−1.63	0.1041	−0.0167	0.00156
Maternal age	0.01645	0.01941	0.85	0.397	−0.0217	0.05457
Married	−0.0194	0.3412	−0.06	0.9547	−0.6896	0.65073
Not married or living with a partner *	0					
Education	−0.0003	0.20943	0	0.9988	−0.4117	0.41103
Creole	−0.2158	0.32541	−0.66	0.5075	−0.8549	0.42333
Hindustani	0.15494	0.32106	0.48	0.6296	−0.4756	0.78553
Indigenous	−0.2682	0.53001	−0.51	0.613	−1.3092	0.77275
Javanese	−0.1814	0.44636	−0.41	0.6847	−1.058	0.69533
Tribal	−0.5713	0.35362	−1.62	0.1067	−1.2658	0.12322
Mixed *	0					
Household income SRD 800+	−0.6863	0.32129	−2.14	**0.0331**	−1.3173	−0.0552
Household income <SRD800 *	0					
0–1 previous live births	0.05431	0.2554	0.21	0.8317	−0.4473	0.55594
2+ previous live births *	0					
Moderate anemia	0.12741	0.24467	0.52	0.6028	−0.3531	0.60795
Mild anemia	0.25597	0.27864	0.92	0.3587	−0.2913	0.80324
No anemia *	0					
Pb × Se	0.00046	0.00022	2.09	**0.0373**	2.7 × 10^−5^	0.00089
Hg × Se	−0.0001	0.00014	−0.88	0.3784	−0.0004	0.00015

* = reference category. Bold indicates significance.

## Data Availability

The original contributions presented in this study are included in the article. Further inquiries can be directed to the corresponding authors.

## Abbreviations

The following abbreviations are used in this manuscript:

ASGM	Artisanal and Small-Scale Gold Mining
BSID-III	Bayley Scales of Infant and Toddler Development, Third Edition
BSID-III-NL	Bayley Scales of Infant and Toddler Development, Third Edition, Dutch Version
CCREOH	Caribbean Consortium for Research in Environmental and Occupational Health
Cd	Cadmium
CVAFS	Cold Vapor Atomic Fluorescence Spectrometry
Hb	Hemoglobin
Hg	Mercury
ID	Intellectual Disability
IQR	Interquartile Range
LMIC/LMICs	Low- and Middle-Income Country/Countries
Mn	Manganese
PAHO	Pan American Health Organization
Pb	Lead
Se	Selenium
SRD	Surinamese Dollar
SPSS	Statistical Package for the Social Sciences
WHO	World Health Organization
